# Carcinogenic mechanisms of virus-associated lymphoma

**DOI:** 10.3389/fimmu.2024.1361009

**Published:** 2024-02-28

**Authors:** Ying Zhang, Wei Guo, Zhumei Zhan, Ou Bai

**Affiliations:** Department of Hematology, The First Hospital of Jilin University, Changchun, Jilin, China

**Keywords:** virus, lymphoma, pathogenesis, EBV, HBV, HCV, HIV

## Abstract

The development of lymphoma is a complex multistep process that integrates numerous experimental findings and clinical data that have not yet yielded a definitive explanation. Studies of oncogenic viruses can help to deepen insight into the pathogenesis of lymphoma, and identifying associations between lymphoma and viruses that are established and unidentified should lead to cellular and pharmacologically targeted antiviral strategies for treating malignant lymphoma. This review focuses on the pathogenesis of lymphomas associated with hepatitis B and C, Epstein-Barr, and human immunodeficiency viruses as well as Kaposi sarcoma-associated herpesvirus *to* clarify the current status of basic information and recent advances in the development of virus-associated lymphomas.

## 1 Introduction

The most consistent risk factors for malignant lymphoma comprise immune dysfunction and infectious agents that are primarily viruses. The concept of virus-induced lymphoma is not new, because viruses are associated with ~ 15% of all types of cancer (1). The pathogenesis of virus-associated lymphoma is complex and involves viral infection, immune disorders or deprivation of immunity, the tumor microenvironment (TME), and several viral co-infections. The complex biological properties of the virus itself, a delicate balance between viral and host immunity, and difficulties with establishing animal models have hindered research and understanding of the pathogenesis of virus-associated lymphoma. Lymphoma-associated viruses are very diverse (**Table 1**). Examples are large double-stranded DNA genomes (Epstein-Barr virus, EBV; Kaposi sarcoma-associated herpesvirus, KSHV), small double-stranded DNA genomes (hepatitis B virus; HBV), and positive-sense single-stranded RNA genomes (hepatitis C virus; HCV). Sufficient evidence indicates that human immunodeficiency virus (HIV), EBV and KSHV are pathogenic factors in lymphoma. However, other evidence indicates a possible relationship between HIV and viruses that cause hepatitis (HBV and HCV) and might be more limited and indirect than EBV and KSHV (2–4). Overall, general pathogenic mechanisms for the development of virus-associated lymphoma have been identified. Viruses can directly infect and transform lymphocytes, and viral antigen products or soluble factors induce chronic B-cell activation and promote transformation. Long-term immunodeficiency, such as that caused by HIV, facilitates viral evasion of the immune response and leads to tumor cloning. Current options for treating virus-associated lymphoma include radiotherapy, chemotherapy, immunotherapy, as well as antiretroviral, antiviral, and targeted therapy. Nevertheless, most virus-associated lymphomas are typically more chemoresistant and have a poorer prognosis than solid tumors. Therefore, a deeper understanding of the molecular mechanisms of virus-associated lymphoma will provide directions to develop targeted therapies.

**Table 1:** Human viruses that are associated with lymphoma and other diseases.

Virus	Gene	Other disease	Lymphoma	Lymphoma in experimental animal model
**HBV**	Small double-stranded DNA genomes	Hepatitis, cirrhosis, Hepatocellular carcinoma	Various NHL, HL (Controversial)	Unknown
**HCV**	Positive-sense single-stranded RNA genomes	Hepatitis, cirrhosis, Hepatocellular carcinoma	Various B-cell NHL	Yes
**HIV**	Retroviruses	Opportunistic infection, malnutrition, Kaposi sarcoma	DLBCL, BL, PEL, PBL, plasmablastic lymphoma of the oral cavity, HL, PCNSL, MCD-associated large cell lymphoma	Unknown
**EBV**	Large double-stranded DNA genomes	Infectious mononucleosis, oral hairy leukoplakia	BL, HL, PTLD, DLBCL, PCNSL, NK/T cell lymphoma,	Yes
**KSHV**	Large double-stranded DNA genomes	Kaposi sarcoma	PEL, MCD, large B-cell lymphoma (NOS), GLPD	Yes

BL, Burkitt lymphoma; DLBCL, diffuse large B-cell lymphoma; GLPD, germinotropic lymphoproliferative disorder; HL, Hodgkin lymphoma; MCD, multicentric Castleman disease; NHL, non-Hodgkin lymphoma; NOS, large B-cell lymphoma not otherwise specified; PCNSL, primary central nervous system lymphoma; PEL, primary exudative lymphoma; PTLD, post-transplant lymphoproliferative disorder.

## 2 Epstein-Barr virus

Epstein-Barr virus (EBV) is the most prevalent human oncovirus (5), and > 90% of adults are infected during their lifetime (6). The main mode of transmission of EBV is through oral transmission via saliva, and the current study confirms that the main tropism of EBV is for B cells and epithelial cells, and the presence of EBV has been demonstrated in tumor cells derived from NK/T cells and leiomyosarcoma (7). When EBV was first isolated from a Burkitt lymphoma (BL) cell line in 1964 (8), its association with cancer was widely studied. According to the 2016 WHO classification, EBV is associated with lymphomas, including mature B-cell tumors, mature T-cell and Natural killer (NK)-cell tumors, Hodgkin lymphoma (HL), and post-transplant lymphoproliferative disorders (9). The prognosis is worse for patients with HL and diffuse large B-cell lymphoma (DLBCL) who are EBV^+^ than EBV^-^. (10) NK/T-cell lymphoma (NKTCL), a rare subtype of EBV-associated non-Hodgkin lymphoma (NHL), has similarly shown poorer outcomes (11).

### 2.1 Epstein-Barr virus structure

Epstein-Barr virus (also known as human herpesvirus 4; HHV-4), belongs to the gamma herpesvirus family. The EBV virion has a diameter of 150-170 nm and consists of a lipoprotein capsule and an icosahedral nucleocapsid, including 162 capsid particles. The viral genome comprises double-stranded DNA of ~ 170 kb. This virus is permanently latent in lymphocytes, free in the cytoplasm as circular DNA and can integrate into cellular chromosomes (12). The life cycle of EBV is biphasic, with lytic replication and a latent phase, and the usual progression of EBV latency in B cells from type III to types II to I has been detailed in a review (13). After infecting resting naïve B cells, EBV enters type III latency, when all latency genes are expressed. The production of highly immunogenic viral proteins triggers a powerful cytotoxic T cell response. Subsequently, the virus restricts gene expression and enters type II latency by expressing Epstein-Barr nuclear antigen (EBNA)-1, latent membrane protein (LMP)-1, and LMP-2. B cells differentiate into memory B cells during this phase. Finally, EBV restricts gene expression to latency type I, where only EBNA-1 and EBV-encoded small RNAs (EBERs) are expressed (14). **Table 2** shows EBV gene expression during various latent infections.

**Table 2:** EBV viral gene expression during different types of latent infection.

Genes	Latency 0	Latency I	Latency II	Latency III
EBNA1	–	+	+	+
EBNA2	–	–	–	+
EBNA3s	–	–	–	+
EBNA-LP	–	–	–	+
LMP1	–	–	+	+
LMP2A	–	–	+	+
LMP2B	–	–	+	+
EBERs	+	+	+	+
BHRF1miRNAs	–	–	–	+
BARTs miRNAs	+	+	+	+

Represent positive and negative gene expression is shown as + and –, respectively.

### 2.2 Carcinogenic mechanisms of EBV

The range of EBV-associated lymphomas is extraordinarily broad, and each has unique developmental pathways. Differences in EBV gene expression among them reflect the different pathogenic roles of EBV. Despite the current scale of research into the relationship between EBV and lymphoma, the etiological role of EBV is difficult to explain. This is partly because the virus acts differently on various tumors and partly because current disease models do not adequately replicate subtle changes in the virus-host balance among EBV-associated cancers. Moreover, although 95% of adults are persistently infected with EBV, most do not develop EBV-associated lymphomas. Therefore, the virus does not act alone, which warrants further exploration. Therefore, we would like to further summarize the mechanism of EBV-associated lymphoma from the perspective of the virus itself.

#### 2.2.1 Expression of viral protein

Latent proteins are essential for the transformation of normal B lymphocytes into lymphoblastoid cell lines (LCLs), and they are involved not only in driving the overexpression of oncogenes, the silencing of tumor suppressors, the cell cycle, migration, but also in the regulation of adhesion.

##### 2.2.1.1 EBNA1

EBNA1 is the only consistently expressed viral protein during the latent phase of EBV, and it is indispensable for the propagation and propagation of the latent viral genome. The current study finds that EBNA1 has significant pleiotropic effects, (15) including disruption of p53 stability (16–18) and promyelocytic leukemia (PML) nuclear bodies (19), and EBNA1 also affects several currently known signaling pathways involved in cell proliferation and apoptosis, known to include interference of EBNA1 with TGF-β signaling (20, 21) and inhibition of NF-κB activity. (22) Moreover, previous studies have also found that stable or transient infection with EBNA1 leads to oxidative stress, allowing reactive oxygen species accumulation and has a variety of effects on cell growth and survival, involving the induction of apoptosis as well as DNA damage. (23, 24) In particular, EBNA1 can co-immunoprecipitate with Nm23-H1 in lymphocytes, which may contribute to the spread of EBV-associated tumors (25, 26). In fact, EBNA1 is actually highly antigenic, and T cells targeting ENBA1 are present in infected individuals (27). Therefore clarifying the immunomodulatory role of EBNA1 for the host has long been a focus of attention for researchers, which has been comprehensively summarized in a recently published review (28). Most published studies have now been limited to immune evasion or immunosuppression (28), include that EBNA1 can specifically bind to viral and cellular DNA for sequences (29–31) and can also enhance and inhibit the transcription of viral and cellular genes (32, 33), and mediate the maintenance of the EBV genome (34). Recent studies have confirmed the trans-immune evasion ability of EBNA1. EBNA1 can inhibit the expression of these genes and enhance the survival and proliferation of infected cells by binding to DNA near the transcriptional start site of NKG2D ligand and c-*Myc* gene (35). In another study (36), EBNA1 was found to target c-*Myc* by chromatin immunoprecipitation (ChIP) sequencing of endogenous bromodomain-containing protein 7 (BRD7) in Burkitt lymphoma(BL), thereby regulating the viral infection status by coordinating with host BRD7. In addition, other studies have found that the expression of Galectin-9 (Gal-9) is positively regulated by EBNA1 at both the mRNA and protein levels (37), and Gal-9 has been shown to be a ligand for immune proteins on immune cell subpopulations and is also involved in cell proliferation and differentiation (38).

##### 2.2.1.2 EBNA2

Many of the virus’ latent genes are expressed in currently established EBV-infected cell lines. Of high interest, Pich et al. (39) explored in depth the first 8 days of infection by using EBV derivatives with a single mutation in EBV and found that EBNA2 played and its important role in activating naïve human B lymphocytes, inducing growth, and facilitating division, and in particular EBNA2 prevented the death of a subpopulation of infected cells. However, EBNA-LP, LMP2A, and miRNAs only have supportive and auxiliary functions. Even EBNA1, which has been in the spotlight, seems to be nonessential for cell activation in early viral infection. Previously known studies have extensively explored the mechanism of action of EBNA2, which is not only a potent activator of transcription of genes such as CD23 (40) and *C-myc* (41), but also negatively regulates genes such as *BCL6* and *lg* (42). Of interest is the previous finding that restricted expression of EBV latent genes contributes to viral persistence by down-regulating the plasma cell master regulator Blimp1, which induces and maintains the mature B-cell phenotype (43). EBNA2 is also a functional homologue of activated Notch (44), while both C-*myc* and activated Notch have oncogenic properties. In a recent study by Zhang et al. (45) it was demonstrated that LMP1 and EBNA2 constitute the minimum EBV proteins required for B-cell transformation, emphasizing the important role of EBNA2 in B-cell transformation, even though the study did not provide an in-depth investigation of the mechanism. EBNA2 is involved in host immunomodulation through its regulation of miRNAs. In B-cell lymphoma, EBNA2 positively regulates miRNA-21 and negatively regulates the expression of miRNA-146a, which affects the antiviral response of the innate immune system and is involved in EBV-induced B-cell transformation. The detailed mechanism has not been published up to now. The study by Anastasiadou et al. (46) found that EBNA2 down-regulated miRNA-34 by recruiting early B-cell factor 1 (EBF1) to the promoter and increased PD-L1 expression in BL and DLBCL. Other research found that EBNA2 also reduces ICOSL expression by inducing miRNA-24 while maintaining pro-proliferative C-*myc* levels to evade host immune responses (47).

##### 2.2.1.3 EBNA-LP

Current studies on EBNA-LP are limited. Like EBNA2, EBNA-LP is also expressed early in infection, and EBNA-LP acts mainly as a co-activator of EBNA2 and participates in B-cell transformation by activating viral and cellular transcription (48). In addition, some studies have demonstrated other effects of EBNA-LP. These include regulation of specific alternative splicing (49), promotion of transcription factor recruitment, and involvement in cell growth and survival (50).

##### 2.2.1.4 EBNA3

The EBNA3 family, consisting of the EBNA3A, EBNA3B, and EBNA3C genes, is thought to be a nonredundant family of EBV genes that likely arose from gene duplication during the evolution of primate gamma herpesviruses (51). The production of EBNA3 proteins is thought to be tightly regulated and, because of their low protein levels and turnover efficiency, these proteins are very stable (52). Interestingly, the EBNA3 family has conflicting roles in carcinogenesis. EBNA3A and EBNA3C promote carcinogenesis, whereas EBNA3B inhibits carcinogenesis (53). EBNA3A stimulates cell proliferation by inhibiting p21^WAF/CIPI^, targeting tumor suppressor pathways and altering cell cycle regulation (54). The mechanisms by which EBNA3C promotes lymphoma development are more diverse, including regulation of cyclin D2 (55) and targeting of tumor suppressor pathways (53). The role of EBNA3 family proteins in EBV-associated B-cell lymphomagenesis has been systematically described (51). Numerous synergistic collaborations between the EBNA3 protein families have been recognized, mostly involving cooperation between EBNA3C and EBNA3A or EBNA3B. Only in the absence of EBNA3C is there moderate cooperation between EBNA3A and 3B. The cooperation between the EBNA3 protein families has been described in detail in the review by Styles et al. (56).

##### 2.2.1.5 LMP1

Among the proteins expressed during EBV viral latency, LMP1 has been of great interest, which is expressed in HL, DLBCL, and post-transplant lymphoproliferative disorder(PTLD) (57, 58), and is essential for the transformation of viral B cells into lymphoblastoid cell lineages, which has been meticulously reviewed in many previous studies (59) (60, 61) (62). The oncogenic mechanism of LMP1 in EBV-associated lymphomas is very complex. EBV not only promotes oncogenic pathways such as Janus kinase/signal transducer, nuclear factor-κB (NF-κB), phosphatidylinositol-3-kinase/protein kinase B (PI3K/AKT), mitogen-activated protein kinase (MAPK), and transcriptional activator of transcription (JAK/STAT) (63), but also, because of its own weaker immunogenicity, it can bypass the targeting effect of CD8^+^ T-cells and fail to elicit an appreciable immune response in EBV-positive healthy people (62). More importantly, LMP1 was associated with increased expression of PD-L1 in a variety of lymphomas (64), which provided new clues to further explore the immunomodulatory role of LMP1. The latest study by Giehler et al. (65) demonstrates a direct protein-protein interaction between LMP1 and TNF receptor-associated factor 6 (TRAF6), which underlies C-terminal activation region 2 (CTAR2) signaling and the survival of LMP1-transformed B-cells, resolving what we have always wondered.

##### 2.2.1.6 LMP2A and LMP2B

LMP2A is expressed in various B-cell malignancies, including HL, PTLD, and BL, but our current studies on the mechanism by which LMP2A promotes lymphomagenesis are not in-depth. Using transgenic mice, Fish et al. (66, 67) demonstrated that LMP2A accelerated lymphoma development *in vivo* by exploiting the role of MYC in the cell cycle, particularly during p27^kip1^ degradation. The latest study utilized phosphoproteomics and transcriptomics to further explore the molecular mechanisms by which LMP2A affects B-cell biology, and found that LMP2A down-regulates cyclic checkpoint genes, including CDKN1B(p27) and CHEK1, as well as the tumor suppressor RB1 (68).

The function of LMP2B is largely unknown. Earlier studies demonstrated that LMP2B negatively regulates the function of LMP2A to prevent the transition from latent to lytic EBV replication (69). In addition, LMP2B affects epithelial cell behavior, such as cell adhesion and motility (70).

#### 2.2.2 Genetic instability

Genetic instability is one of the major common features of cancer and can be observed at the chromosomal or genetic level in malignant cells (71). Integration of EBV into the host genome may be a common occurrence in lymphomas, but our understanding of this is limited. On the one hand, the large size of the EBV genome itself makes it difficult to determine the integration site with the host genome and to analyze it further, on the other hand, the highly methylated DNA hinders the mapping of the EBV genome, and not only that, multiple copies of the viral exons can generate interference noise at the integration site (72), which makes it more difficult to study it in depth. Previous studies have demonstrated the integration of EBV in the chromosomal genome of BL (73) and other B-cell lymphomas (74, 75). Takakuwa et al. (71) demonstrated in Raji that integration of EBV into 6q15 resulted in loss of expression of the human Bach2 gene (*BACH2*) at the mRNA and protein levels. *BACH2* has been shown to have a significant inhibitory effect on cellular proliferation, and deletion of *BACH2* expression may contribute to the development of B-cell lymphomas, including BL. Related studies have previously analyzed copy number alterations (CNAs) and gene expression profiles of EBV^+^ and EBV^-^DLBCL samples confirming that EBV^+^ DLBCL has fewer genomic alterations (76). In a recent whole-exome sequencing of EBV^+^DLBCL, it was shown that a heterogeneous mutational landscape is associated with DNA double-strand break-homologous recombination repair failure, and genes found to have a high number and frequency of mutations include serine protease 3 (*PRSS3*), *MUC3A* and *MUC16* (77). A recent study by Zhou et al. (78) demonstrated an elevated frequency of mutations in *MYC* and *RHOA* in patients with EBV^+^DLBCL. An updated mutational map of EBV^+^DLBCL has been comprehensively characterized, complementing previous studies with recurrent alterations in *CCR6*, *CCR7*, *DAPK1*, *TNFRSF21*, and *YY1* (79), further elucidating the mechanism by which EBV leads to B-cell transformation.

#### 2.2.3 MicroRNAs

EBV was the first virus to detect viral miRNAs (80). The EBV genome encodes 44 mature miRNAs belonging to two distinct classes, BamHI-A region rightward transcript (*BART*) and Bam HI fragment H rightward open reading frame 1 (*BHRF1*), which have different expression levels in different EBV latency types (81). Among them, *BART* transcripts encode 22 miRNA precursors and 40 mature miRNAs, while *BHRF1* transcripts express three miRNA precursors to produce four mature miRNAs. Current published literature has demonstrated that EBV-encoded miRNAs play an important role in the development and progression of EBV-associated malignancies, including cell proliferation, apoptosis, invasion, and transformation (82, 83).Moreover, EBV miRNAs can even directly target immune-related genes, allowing infected cells to evade surveillance and destruction of the immune system (84), (85). However, EBV miRNAs have different expression profiles in different cancer types. In EBV-infected DLBCL, all EBV-miRNAs except *BHRF1* cluster and EBV-miR-BART15 and -20 could be detected, as demonstrated in Imig et al. And in NK/T-cell lymphomas, the most highly expressed viral miRNAs were miR-BART1-5p, miR-BART5, miR-BART7, miR-BART11-5p, and miR-BART19-3p, accounting for 50% of viral miRNAs and approximately 1% of total miRNAs (86). Studies have described the presence and expression levels of EBV miRNAs and host miRNAs in different lymphomas, with some focusing on patient samples and others on different cell line models for *in vitro* experiments. EBV microRNA profiles and human microRNA profiles for EBV-associated lymphomas are detailed in a recent study by Soltani et al. (87) What’s more, published studies have confirmed that EBV-encoded miRNAs may interfere with host miRNAs, which actually leads to even more complications (87).

EBV miRNAs are essential for regulating the viral life cycle. It was demonstrated as early as lizasa et al. (88) that EBV-miRNA-BART6-5p targets four sites within the 3’-UTR of human Dicer mRNA and comprehensively affects the maturation of the miRNAs, resulting in the total repression of these molecules, which helps to maintain latent infection. Of particular note, in addition to EBV miRNAs, EBV-associated products also contribute to the downregulation of Dicer, such as the EBNA1 protein, which has been described in detail in Mansouri et al. (89) EBV miRNA biogenesis and action are also affected by adenosine to inosine (A-to-I) RNA editing. A-to-I editing of pri-miR-BART6-5p was found in EBV-infected BL to activate Zta and Rta viral proteins encoding *EBNA2* viral oncogenes and essential for lysis and replication, leading to the transition of the viral cycle to type III latency (88). Of interest, EBV-encoded miRNAs are also involved in host cell growth, cell cycle, and apoptosis. *PRDM1/Blimp1* is a major regulator of terminal B-cell differentiation and is well known as an oncogene in aggressive lymphomas. Nie et al. (90) have demonstrated that the cellular target of the EBV-miRNA-BHRF1-2 is *PRDM1*, and that by inhibiting the *PRDM1*-mediated function and conferred a growth advantage to EBV-infected B cells, promoting lymphoma development. Another study confirmed that the EBV-miRNAs-BART9 were involved in the proliferation of Nasal NK/T cell lymphomas (NKTCL) by regulating the level of LMP-1 (91).

**Figure 1:**
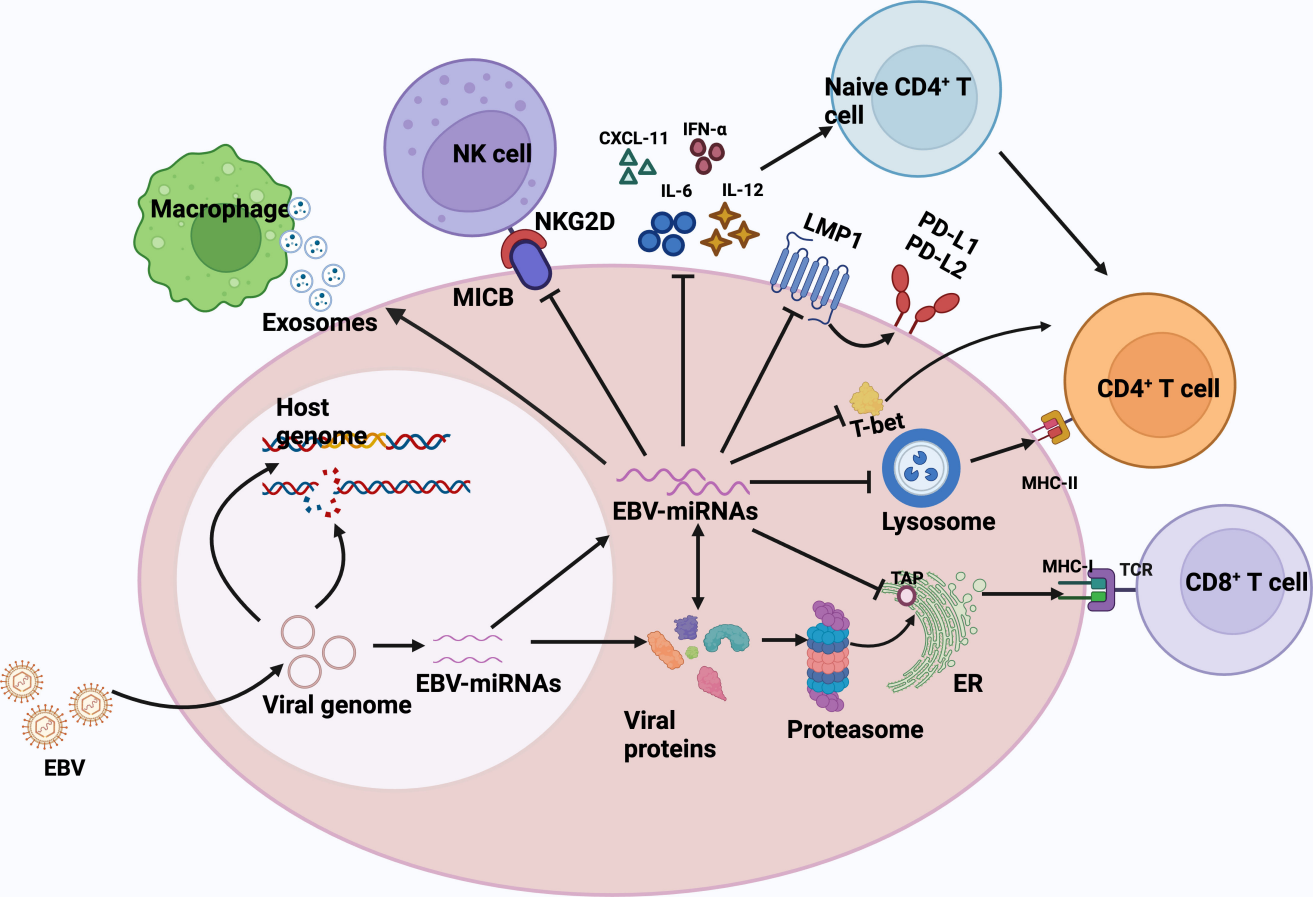
EBV miRNAs are involved in regulating the host immune response. Biogenesis of EBV-encoded miRNAs is dependent on host mechanisms and comprehensively controls the antiviral adaptive immune response of infected B cells. Immediately after infection, the viral DNA genome is circularized and virally encoded and noncoded RNAs are expressed. EBV miRNAs support immune evasion at multiple levels.1) EBV miRNA-BHRF1-2-5p targets the viral antigen LMP1, driving the expression of PD-L1 and PD-L2, and facilitating viral persistence in host cells.2) EBV miRNAs also effectively interfere with MHC class I-mediated antigen presentation by targeting the antigen transporter protein, TAP2. TAP2 is a target of miRNA-BHRF -13 and -BART17.3)EBV miRNA inhibits the expression of lysosomal enzymes (IFI30, LGMN, and CTSB), of which IFI30 and LGMN are under the control of miR-BART1 and -BART2, respectively, and CTSB is controlled by miRNA-BART2 and -BHRF1-2, inhibiting the antigen presentation ability to CD4^+^ T cells via MHC class II.4) EBV miRNA-BART20-5p inhibits T-bet translation by secondary inhibition of p53 and thus inhibits T-bet translation.5) EBV miRNAs also control the expression of inflammatory cytokines (IL-12, IL-6, and IFN-α), thus inhibiting cytokine-mediated immune response.6) miRNA -BHRF1-3 reduces the secretion of the NK cell ligand CXCL-11, allowing infected B cells to evade immunization by NK cells and T cells.7) EBV acts in trans on uninfected macrophages in tumors by secreting exosomes and promotes lymphoma development. CXCL-11,C-X-C motif chemokine ligand 11; ER, endoplasmic reticulum; TCR, T-cell receptor; MHC, major histocompatibility complex; NKG2D, natural killer group 2D; MICB,MHC class I chain-related molecule B.

The success and persistence of any viral infection depends on a complex balance with the host immune system, and EBV miRNAs are also involved in the regulation of the host immune system. (**Figure 1**) EBV-miRNAs-BART6-3p was found to mediate down-regulation of the interleukin-6 receptor (IL-6R) in BL (92), which is involved in regulating key cellular processes, including cell proliferation, survival, and response to host pathogens after dimerization receptor binding to interferon-α, IL-12, or IL-27 (93). In addition, the EBV-miRNAs-BART20-5p were shown to inhibit T-bet translation through secondary inhibition of p53 (94). The role of EBV-encoded miRNAs in immunomodulation has been well and exhaustively described (84, 95–97). The latest research has confirmed that in DLBCL, EBV-miRNA-BHRF1-2-5p targets LMP1 to drive the expression of PD-L1 and PD-L2, exerting context-dependent immune counter-regulation, leading to immune escape and contributing to persistent viral infection (98). In another study, Murer et al. (99) used NOD-SCID _γc_ null (NSG) and HLA-A2 transgenic NSG mice to construct a mice model infected with an EBV variant infection lacking viral miRNAs and a mice model infected with wild-type EBV, which found that the viral load in mice infected with EBV variants lacking viral miRNAs was significantly reduced, and the proliferation frequency of EBV-infected B cells was also decreased. What’s more, the depletion of T CD8^+^ cells led to the formation of lymphomas n the mouse model infected with the viral miRNA-deficient variant, which supports the notion that EBV miRNAs play a major role in immune evasion *in vivo* and support tumor development. The role of EBV virus-encoded microRNAs in human lymphomas can be found in the review by Navari et al. (82).

## 3 Hepatitis B virus

According to the World Health Organization (WHO), 257 million people worldwide have chronic HBV infection defined as hepatitis B surface antigen (HbSAg) positivity. The geographic epidemiological profile of HBV is clear according to the WHO; the prevalence is 6.1% in Africa, the Western Pacific, and Southeast Asia, and 1.6% in Europe and North America. (100) Worldwide, the most common route of transmission of HBV is perinatal, but it can also be transmitted percutaneously and via mucous membranes, as well as through sexual intercourse. (101) When infection occurs, the host may experience acute infection with full recovery, or chronic infection or an acute course leading to hepatic failure. (102) The relationship between HBV infection and NHL has been explored (103–105). However, HBsAg^+^ is not associated with elevated risk of HL, multiple myeloma (MM), or various types of leukemia (106). Compared with HBsAg^-^ DLBCL, the median age of HBsAg^+^ DLBCL onset is younger, with more frequent splenic or retroperitoneal lymph node involvement, more advanced disease, and significantly worse outcomes (107). The results of other studies are similar (106, 108–110). A meta-analysis of 58 studies revealed that HBV infection leads to a 2.5-fold increased risk of NHL, and data from a stratified analysis suggest a closer association between HBV infection and B-cell, than T-cell NHL (111). Why HBV infection is more closely associated with B- than T-cell lymphoma requires elucidation in functional studies.

### 3.1 Hepatitis B virus structure

The hepatitis B virus (HBV) is a prototype that belongs to a family of small, enveloped, hepatotropic DNA viruses that infect a narrow host range of mammals and birds and preferentially orientate towards hepatocytes (112). After HBV infection of hepatocytes, the genome of HBV is delivered into the nucleus and repaired in the nucleus to form covalently closed circular DNA (cccDNA), which is then used as a template to guide the transcription of viral RNA. cccDNA is highly stable in the nucleus of infected hepatocytes, which is why chronic hepatitis B is difficult to treat thoroughly (113). The HBV genome contains four overlapping open reading frames (ORFs), four promoters, two enhancer elements (EN1 and EN2), a polyadenylation site for viral RNA transcription and several *cis*-acting signals for DNA replication. The ORFs P, S, C, and X in the negative strand respectively encode DNA polymerase, HBsAg protein, core and pre-core proteins, and X protein (HBx). (114).

Such DNA viruses have unusual replication features through RNA intermediates and can integrate into the host genome.

### 3.2 Carcinogenic mechanisms

**Figure 2:**
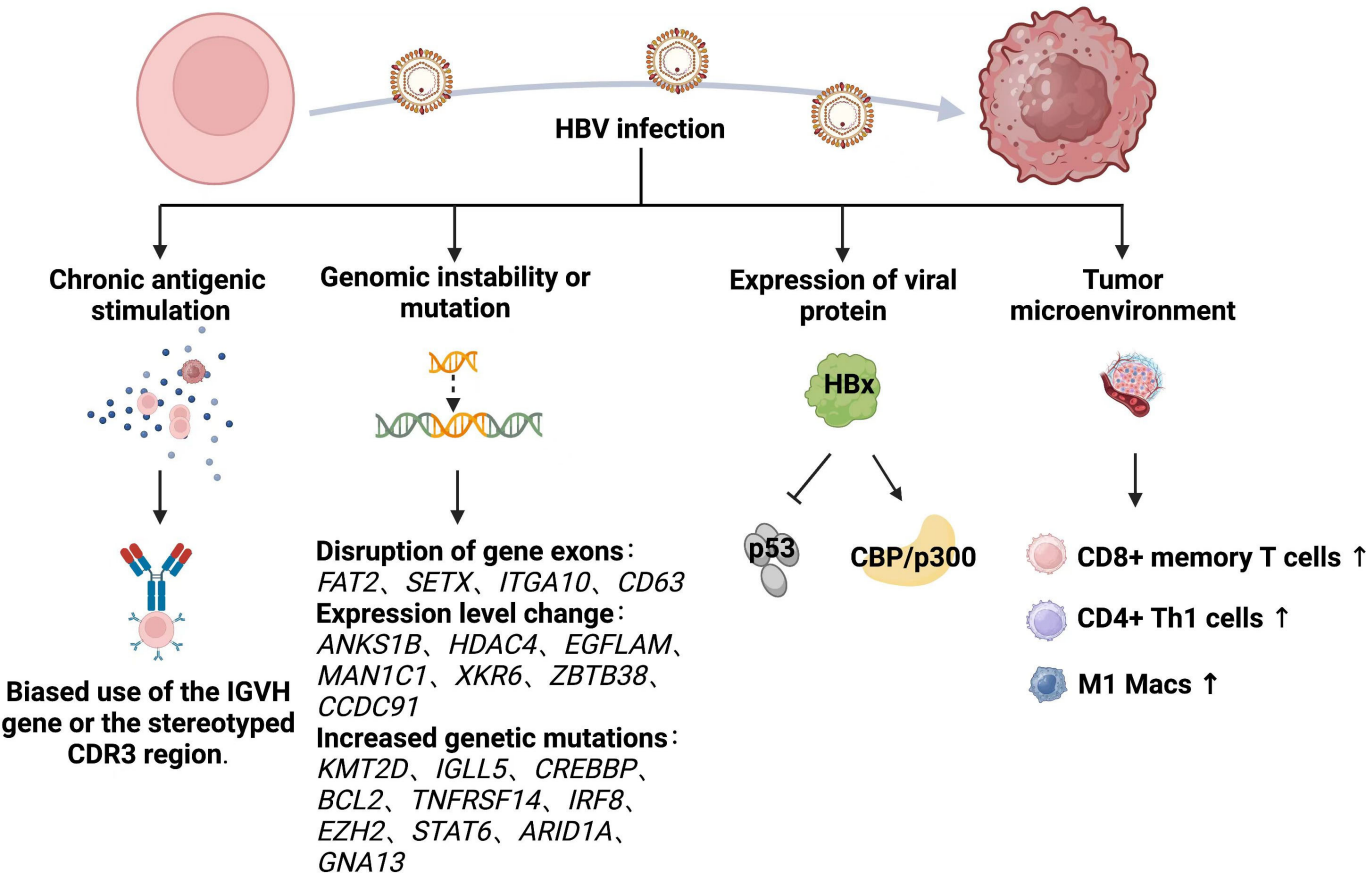
Mechanism of HBV causing lymphoma development.

The biological mechanisms through which HBV infection causes lymphoma are unclear. Those specific to HBV-associated lymphoma have been inferred primarily from studies of HBV-associated hepatocellular carcinoma (HCC) and HCV-associated lymphoma. We emphasize the importance of the humoral and cellular immune systems are important for viral clearance (115), as both are activated by HBV infection and exert antiviral effects. The two immune system might destroy host cells that are already infected with HBV. Therefore, the potential role of HBV in the development of lymphoid disease might be very complex. Various hypotheses have been proposed to explain the mechanisms through which HBV causes lymphoma, and these are summarized below (**Figure 2**).

#### 3.2.1 Chronic antigenic stimulation

The hypothesis that chronic antigenic stimulation causes lymphomas remains controversial. Chronic local antigen-stimulated immune responses caused by HBV infection might be associated with the development of lymphoma (116). A large 14-year follow-up cohort study in Korea (106) consistently associated HBsAg^+^ with elevated risk of NHL, suggesting that chronic infection promotes the development of lymphoma. Risk of B-NHL is not increased in individuals previously infected with HBV or vaccinated against HBV (117, 118). Nucleic acid sequences specific to HBV have been detected in peripheral blood nuclei and hematopoietic tumor cells of patients with HBsAg^+^ (3, 119, 120), which might result in chronically stimulated B cells that transform into B-cell NHL. Peripheral blood mononuclear cells (PBMCs) derived from patients with chronic HBV infection have immortalization potential when cultured *in vitro* (121). New cells identified in the peripheral blood of some patients with non-lymphoid chronical HBV infection were later confirmed as being of B-cell origin. Moreover, the immunophenotype of these cells was similar to that of most HBsAg^+^ B-cell NHL. This supported the relevance of HBV-induced B-cell NHL, although none of the patients developed lymphoma during > 1 year of follow-up. Furthermore, a 42.1% and 65.5% bias towards *I*mmunoglobulin Heavy Variable 4-34 (*IGHV4-34*) heavy, and Immunoglobulin Kappa Variable 4-1 (*IGKV4-1*), light-chain genes respectively in HBsAg^+^ DLBCL, exceeded that in normal peripheral blood B cells and B-cell NHLs (107). However, these results were contradicted by a study that found no evidence of biased *IGVH* gene usage or the stereotyped third complementarity determining region (CDR3) (122). Unlike classical antigen-driven hepatitis C virus-associated lymphoma, the chronic antigen stimulation model seems less applicable to HBV-associated DLBCL.

#### 3.2.2 Genomic instability or mutation

Hepatitis B viral DNA is integrated into the chromosomal DNA of lymph node cells (123). A genome-wide investigation of HBV integration in HCC found that HBV integration alters chromosomal stability and gene expression, and shortens the overall survival of infected individuals (124). Approximately 50% of woodchuck hepatitis virus (WHV) is integrated into the myelocytomatosis oncogene (MYC) family of genes and it affects the proto-oncogene in woodchuck models of HCC with chronic WHV infection (125). In fact, HBV integration is common, occurring in 80%-90% of HBV-associated HCC (126, 127).

Hepatitis B viral DNA can be integrated into the genome of NHL cells, and like HCC, it has preferential targets in NHL, since exons of the protein-coding genes FAT Atypical Cadherin 1 (*FAT2)*, Senataxin (*SETX*), Integrin Subunit Alpha 10 (*ITGA10*) and Granulophysin (*CD63*) are disrupted by HBV DNA and the expression of seven HBV preferential target genes, Ankyrin Repeat and Sterile Alpha Motif Domain Containing 1B (*ANKS1B*), Histone Deacetylase 4 (*HDAC4*), EGF Like, Fibronectin Type III And Laminin G Domains (*EGFLAM*), Mannosidase Alpha Class 1C Member 1 (*MAN1C1*), XK-Related 6 (*XKR6*), Zinc Finger And BTB Domain Containing 38 (*ZBTB38*), and Coiled-Coil Domain Containing 91 (*CCDC91*) is significantly altered in NHL (128). The expression of six of these genes is increased in NHL whereas that of *HDAC4* is not, suggesting that HBV integration leads to the cis-activation of primary oncogenes rather than the inactivation of tumor suppressor genes. However, no evidence of HBV DNA integration into the tumor genome has been found in either HBV-associated FL (129, 130) or DLBCL (122). A trend towards an increased genome-wide mutational load has been identified by whole genome, or whole exon sequencing in the coding regions of HBsAg^+^ follicular lymphoma (FL) with significantly more non-silent mutations per tumor (129). The most significantly mutated genes were Histone-Lysine N-methyltransferase 2D (*KMT2D*), Immunoglobulin Lambda‐Like Polypeptide 5 (*IGLL5*), CREB-binding protein (*CREBBP*), B Cell Lymphoma 2 (*BCL2*), Tumor Necrosis Factor Receptor Superfamily 14 (*TNFRSF14*), Interferon Regulatory Factor 8 (*IRF8*), Enhancer Of Zeste 2 Polycomb Repressive Complex 2 (*EZH2*), Signal Transducer And Activator Of Transcription 6 (*STAT6*), AT-Rich Interaction Domain 1A (*ARID1A*), and *Guanine Nucleotide-Binding Protein Subunit Alpha-13* (*GNA13)*. Furthermore, the most obvious mutational pathways were HBV infection-associated, followed by the Forkhead Box O (FoxO), Wingless/Integrated (Wnt), Janus Kinase/STAT (JAK-STAT), B-Cell Receptor (BCR), Phosphatidylinositol-3 Kinase (PI3K), and Nuclear Factor Kappa B (NF-κB) signaling pathways.

#### 3.2.3 Expression of viral protein

The HBx protein encoded by the X gene was once named “viral oncoprotein.” This protein is involved in hepatocyte transformation through regulation of the cell cycle and the pleiotropic activity of DNA repair and signaling pathways (131–133). The expression of HBV antigens, especially HBx protein, is abundant in HBV^+^ DLBCL sera (103). These findings were consistent with the significantly elevated HBx levels in HCC due to stable HBV integration (124, 134). The HBx protein inhibits p53 in hepatocytes, which leads to abnormal hepatocyte division and HCC (135, 136). A similar B cell mechanism might contribute to the malignant transformation and development of B cell NHL (3). Among the various activities of HBx, its transactivation might play a crucial role in carcinogenesis. Interaction between HBx and the acetyltransferase CREBBP/p300 facilitates the recruitment of these cofactors to the CREB-responsive promoter, which leads to the activation of gene expression (112). A Chinese study of HBV-associated FL found significantly upregulated CREBBP-binding genes in HBsAg^+^, compared with HBsAg^-^ FL (129). This could explain the low dependence of HBsAg^+^ FL on *CREBBP* mutations in that study, as interaction between HBx and CREBBP/p300 might mimic the role of mutant *CREBBP* during the early stages of lymphoma. The contribution of HBx to the pathogenesis of lymphoma remains obscure, and further investigation is needed to verify its mechanism of action.

#### 3.2.4 Tumor microenvironment

The tumor microenvironment is a complex system of cellular and subcellular components with reciprocal signaling pathways that play key roles in carcinogenesis (137). Tumorigenesis is dependent on the TME, and stroma is uniformly and inappropriately activated in cancer, thus contributing to the malignant features of tumors (138). Chronic and persistent HBV infection induces immune cell dysfunction, T-cell failure, as well as the extensive activation and production of numerous cytokines, chemokines and growth factors that constitute a sophisticated TME that might affect cancer development (139, 140). Hepatitis B surface antigen-positive FLs might have an altered TME with increased infiltration of cluster of differentiation (CD)8^+^ memory T cells, CD4^+^ Th1 cells, M1-macrophages and increased T cell failure (129). This was consistent with similar findings in HCC associated with HBV.

The unique biological characteristics of HBV complicates exploring curative mechanisms, and animal models have various strengths and weaknesses. This might explain to some degree, the limited progress of investigations into HBV-related lymphoma.

## 4 Hepatitis C virus

An estimated 71.1 million people worldwide are infected with HCV, with an annual incidence of 1.75 million (141). The most common routes of HCV transmission are blood transfusions, health care-related injections and injecting drug use (142). Most people (75-80%) will develop chronic infection after exposure to HCV, and the clinical cases of acute hepatitis C are less than 25% (142). In addition to infecting hepatocytes, HCV can infect other cells, such as lymphocytes (143). A possible association between HCV infection and NHL was first described in 1994 (144). A study of 150,000 patients with HCV in the USA found that HCV infection increased risk of lymphoma by 20%–30% (145). Epidemiological data show no, or only a slight increase in the risk of T-cell NHL and HL (146, 147), while the strongest evidence is for B-cell NHL (148). A meta-analysis found that the prevalence of HCV infection in patients with B-cell NHL is ~ 15% (149), and others have reached similar conclusions (150–152). We found that the histological subtypes of NHL most closely associated with HCV infection were marginal zone lymphoma (MZL), lymphoplasmacytic lymphoma, and DLBCL (153–156). Clinical HCV^+^ NHL usually occurs after infection for >15 years (157) and patients with HCV^+^ DLBCL usually have higher International Prognostic Index (IPI) scores and LDH levels (158, 159).

### 4.1 Hepatitis C virus structure

The life cycle of HCV begins with the binding of HCV to specific entry factors on hepatocytes, after which the virus is internalized into the cytoplasm. Subsequently, its genomic RNA is released and used for multiprotein translation and viral replication (143). The small, enveloped, positive-sense, single-stranded RNA HCV belongs to the *Flaviviridae* family of the genus *Hepatophilus*. The icosahedral diameter of the envelope particles is 56-65 nm (160), whereas that of the viral core is ~ 45 nm (161). The HCV genome is a positive single-stranded RNA comprising ~ 9,600 nucleotides. It encodes a single open reading frame (ORF) flanked by five and three untranslated regions (UTRs). The HCV polyprotein encoded by a single ORF is ~ 3,000 amino acids long and undergoes co-translational and post-translational processing by cellular and viral proteases to form three structural proteins (core, E1, and E2), an ion channel protein (p7), and the nonstructural (NS) proteins, NS2, NS3A, NS4A, NS4B, NS5A, and NS5B. The structural and NS proteins are located at the N-terminus, whereas other proteins are located at the C-terminal end (162).

### 4.2 Carcinogenic mechanisms

**Figure 3:**
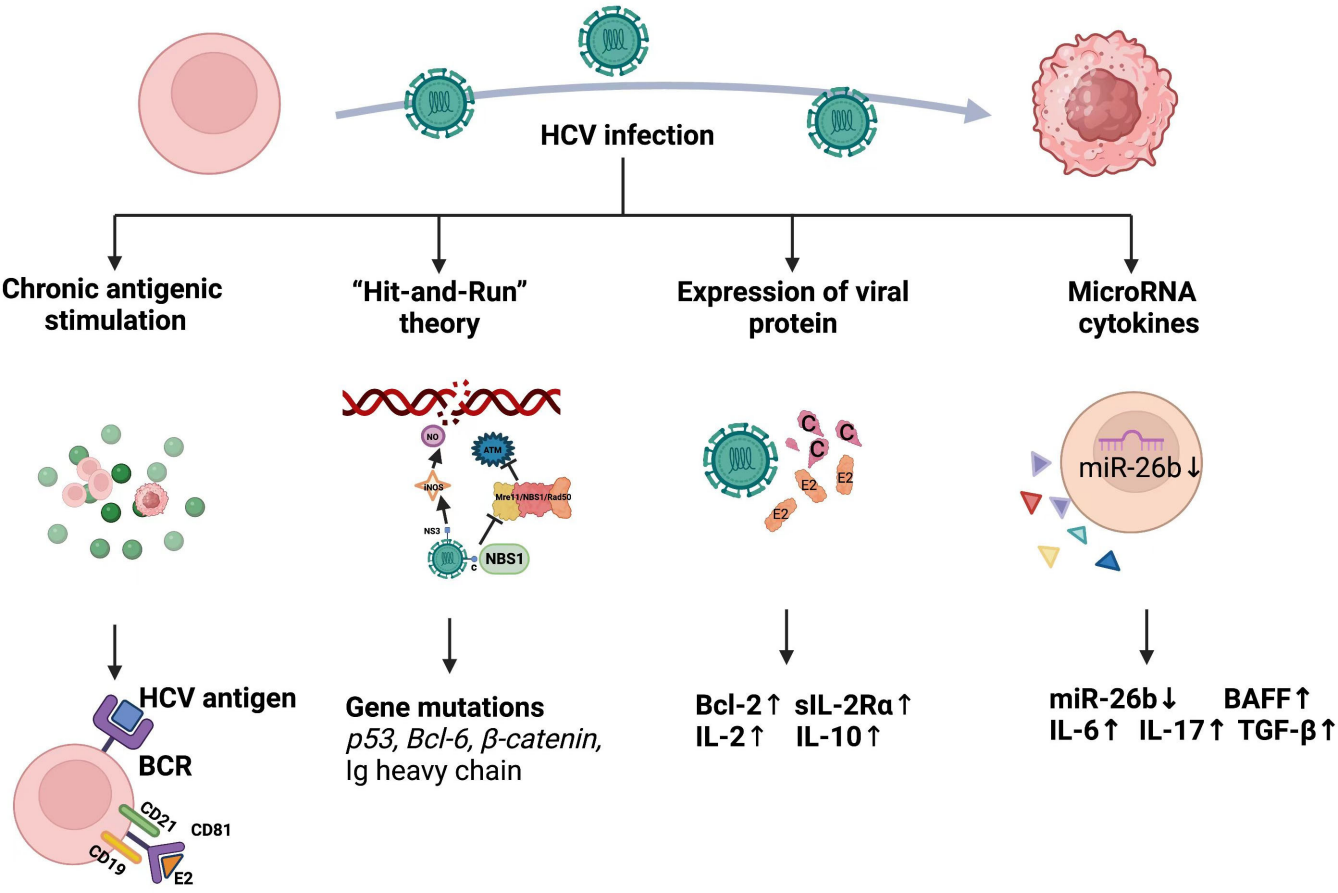
Mechanism of HCV causing lymphoma development.

The integration of single-stranded RNA into HCV nucleic acid sequences of the host genome appears to be impossible owing to the absence of a reverse transcriptase. Therefore, it indirectly exerts oncogenic effects by modulating the host immune system (163). Liver cells and lymphocytes share the HCV receptor, CD81 (164, 165). Activation-mediated CD81 differs from other B cell stimuli because it induces the preferential proliferation of naïve B cells. Expression of the C-X-C Motif chemokine receptor 3(CXCR3) is upregulated in CD81-activated B lymphocytes, but decreased when stimulated with different substances (166). This interaction between HCV and the immune system might underlie the immune and lymphoid tissue proliferative disorders that frequently accompany chronic HCV infections. Three theories might explain HCV transformation (**Figure 3**).

#### 4.2.1 Chronic antigenic stimulation

The defined pathogenic link between chronic *Helicobacter pylori* infection and the development of mucosa-associated lymphoid tissue (MALT) gastric lymphoma suggests that chronic antigenic stimulation can determine the likelihood of NHL (167). Notably, the regression of MALT lymphoma after HP eradication makes this possibility more plausible (168). Splenic lymphoma regression after antiviral therapy similarly eradicates HCV (169). About 10% of patients with type II mixed cryoglobulinemia (MC) develop overt B-NHL after 5-7 years of follow-up (170), and HCV is a major etiological factor in MC and might also be the cause of its evolution to overt NHL (171–173). HCV-associated type II MC expresses immunoglobulins encoded mainly by germline V_H_1-69 and VκA27 genes. A preference for the V_H_1-69/VκA27 combination in HCV-associated lymphomas is consistent with the possible role of antigen selection in the expansion of B cell clones (174). In addition, B-cell receptors expressed by lymphomas in patients infected with HCV rarely react with viral proteins (175). Notably, the highly biased stereotyped BCR sequence of HCV^+^ B-NHL has also been found in other HCV-B-cell malignancies (176). This confirmed that HCV-associated lymphoma cells originate from precursors with autoimmune properties rather than from B cells that express antiviral BCR.

The HCV envelope protein E2 can bind to CD81 expressed on B cells (164). This receptor is upregulated in HCV infection and MC and positively correlates with viral load (177). Moreover, CD81 forms a conjugate complex with CD19 and CD21 in human B cells (178, 179), and the attachment of the B cell antigen receptor (BCR) to any component of this complex decreases the threshold required for BCR-mediated B-cell proliferation (180). Bound E2-CD81 is also involved in activating the transcription factor NF-κB, which subsequently increases the expression of Bcl-2 protein, thus enhancing B cell survival and protecting human B lymphocytes from Fas-mediated apoptosis (181). In addition, HCV E2 binds to CD81 antibodies on neonatal human B cells, which leads to the activation and sequential proliferation of the C-JUN N-terminal kinase pathway (166). Furthermore, HCV-E2 binding to CD81 directly prevents the functional activation of NK cells, providing an effective immune escape strategy for the virus (182). Overall, the interaction between HCV and CD81 promotes chronic infection and facilitates the development of HCV-associated B-cell lymphoma.

#### 4.2.2 Hit-and-run theory

Some evidence indicates that intracellular viral replication is not required for tumor transformation (183). The hit-and-run theory suggests that viruses play a predisposing role in cancer formation and that the viral genome can be completely lost after the host cell has accumulated numerous mutations (184). This mechanism was suggested for HCV (185). Infection with HCV results in a 5-10-fold increase in the frequency of mutations in the Ig heavy chain, B cell Lymphoma 6 (*BCL-6*), Protein 53 *(p53)* and Catenin genes in HCV-infected B-cell lines and HCV-associated peripheral blood mononuclear cells, lymphomas, and HCC *in vitro*. The authors concluded that HCV induces a mutator phenotype by causing changes in proto-oncogenes and oncogenes that successively lead to oncogenic B cell transformation, even when the virus might have already left the cells. The same group also conducted RNA interference experiments and found that HCV induced error-prone DNA polymerases ζ, ι, and activation-induced cytidine deaminase. All these together contribute to increased mutation frequency, complementing the oncogenic mechanism of HCV causing lymphoma. Some controversy remains regarding the clinical applicability of these findings, as they have not been confirmed *in vivo* (186, 187).

Infection with HCV stimulates nitric oxide (NO) production by activating the inducible NOS (iNOS) gene through the viral core and NS3 protein (188). Nitric oxide causes DNA breaks and enhances DNA mutations. The HCV core protein binds to NBS1 and inhibits formation of the Mre11/NBS1/Rad50 complex, thus affecting Ataxia Telangiectasia-Mutated (ATM) activation and inhibiting DNA binding by repair enzymes (189). Infection with HCV inhibits multiple DNA repair processes and leads to chromosomal instability, which explains its oncogenicity from a different perspective.

#### 4.2.3 Expression of viral protein

Hepatitis C viral RNA and protein were detected in an established HCV-infected B-NHL cell line *in vitro* using RNase protection assays and immunoblotting (190). That study confirmed that HCV can infect primary human hepatocytes, PBMCs and established Raji B cell lines *in vitro*, indicating that HCV can replicate in B cells. Ample evidence supports the notion that intracellular viral proteins contribute to oncogenic transformation. Interferon regulatory factor-1-null (irf-1(-/-)) mice with inducible and persistent expression of HCV structural proteins (irf-1/CN2 mice) have been established (191). These mice have a high incidence of lymphoma and lymphoproliferative disorders. The HCV core and E2 proteins are responsible for the expression of interleukin (IL)-2, -10, and -12, as well as the induction of Bcl-2 in the presence of nucleocapsid proteins in the context of complex signaling networks in these mice (191). Another transgenic mouse model expressing HCV core protein frequently developed follicular center cell-type lymphoma, and HCV core mRNA was detected in lymphoma tissues (192). Transgenic RzCD19Cre mice express the complete HCV genome in B cells (193). However, the incidence of DLBCL in RzCD19Cre mice was only 25%. The incidence of B-cell lymphoma correlated significantly with serum levels of soluble interleukin-2 receptor α subunit (sIL-2Rα) only in the RzCD19Cre mice.

#### 4.2.4 MicroRNA and cytokines

Small non-coding MicroRNAs (miRNAs) sequence-specifically regulate gene expression at the post-transcriptional level (194). They play roles in controlling various biological functions such as developmental patterns, cell differentiation, proliferation, genomic rearrangement and transcriptional regulation (195). MicroRNA-26b is significantly downregulated (*P* = 0.0016) in HCV^+^ splenic marginal zone lymphoma (SMZL) and this might cause miR-26b to stop inhibiting never in mitosis gene A (NIMA)-related Kinase 6 (NEK6) and have oncogenic potential in HCV-associated SMZL (196). MicroRNA-26b functions not only in the specific area of HCV-associated SMZL, but also in HCV-associated NHL, including MZL and DLBCL (197). Overall, these findings suggest that miRNA network dysregulation is involved in the development of HCV-associated lymphomas.

Cytokines are small glycoproteins and peptides that usually have relatively short half-lives and act via autocrine and paracrine signaling. Cytokines mediate interactions between immune and non-immune cells in tumors and can promote or inhibit cancer cell growth (198). B-cell activating factor (BAFF) is a key survival factor for B cells that is upregulated during HCV infection (199). An excess of BAFF in the absence of protective tumor necrosis factor (TNF) leads to a high incidence of lymphoma in BAFF transgenic mice, suggesting that BAFF functions in promoting B-cell malignancy (200). Notably, other cytokines and growth factors, including IL-6, -17, -10 and TGF-ß, also contribute to B-cell proliferation in HCV infection (201–203).

However, the molecular mechanisms underlying the development of HCV-associated lymphomas remain poorly understood. The prevailing views are not mutually exclusive and might involve parallel pathways leading to HCV-associated lymphoma, as it is likely that a combination of translational conditions is required to eventually lead to the development of lymphoma. Additional bridging studies combining *in vivo* and *ex vivo* investigations are required to further explore this topic.

## 5 Human immunodeficiency virus

It is estimated that 38.6 million people are currently infected with HIV-1 worldwide, that some 25 million people have died, and that heterosexual transmission remains the dominant mode of transmission, accounting for about 85 per cent of all HIV infections (204). HIV infection carries multiple immune cell types for CD4 and CXCR4/CCR5 co-receptors. This includes helper T cells, macrophages. If untreated, it may also infect microglia and astrocytes of the nervous system (205).An association between HIV and aggressive lymphoma was initially reported in 1982 (206). As the most prevalent malignancies among patients infected with HIV, the relative risks of NHL and HL are 60-200- and 8-10-fold higher than patients with lymphoma without HIV infection, respectively (207, 208). The WHO classification system recognizes subtypes of HIV-NHL (9b). Over 95% of malignancies are of B-cell origin, including DLBCL and BL, whereas plasmablastic, T-cell, and primary effusion lymphomas, are rare, and primary central nervous system (CNS) lymphoma is a very rare B-cell subtype that was more prevalent during the early stages of the AIDS epidemic. These lymphomas have high-grade features such as typically late presentation, extra-nodal involvement, and a marked tendency to involve the gastrointestinal tract, CNS, liver, bone marrow and perinodal soft tissues (209). Despite the introduction of highly active antiretroviral therapy (HAART) and the improved survival rates of patients infected with HIV during the past 20 years, malignant lymphoma remains the leading cause of morbidity and mortality (210).

### 5.1 Human immunodeficiency virus structure

The two types of HIV isolates comprise types 1 (HIV-1) and 2 (HIV-2). The globally predominant pathogen of AIDS is HIV-1, whereas HIV-2 is restricted to certain areas of West and Central Africa (211).Human immunodeficiency virus forms spherical, membrane-enveloped, pleomorphic virions, 1,000–1,500 Å in diameter. that contain two copies of a single-stranded, positive-sense RNA genome (212) This virus is characterized by the structural genes *gag*, *pol*, *env* (211). Like other retroviruses, *gag* genes encode the structural proteins of the core (p24, p7, and p6) and matrix (p17), and *env* genes encode the viral envelope glycoproteins gp120 and gp41. The *pol* encodes enzymes that are essential for viral replication.

### 5.2 Carcinogenic mechanisms

**Figure 4:**
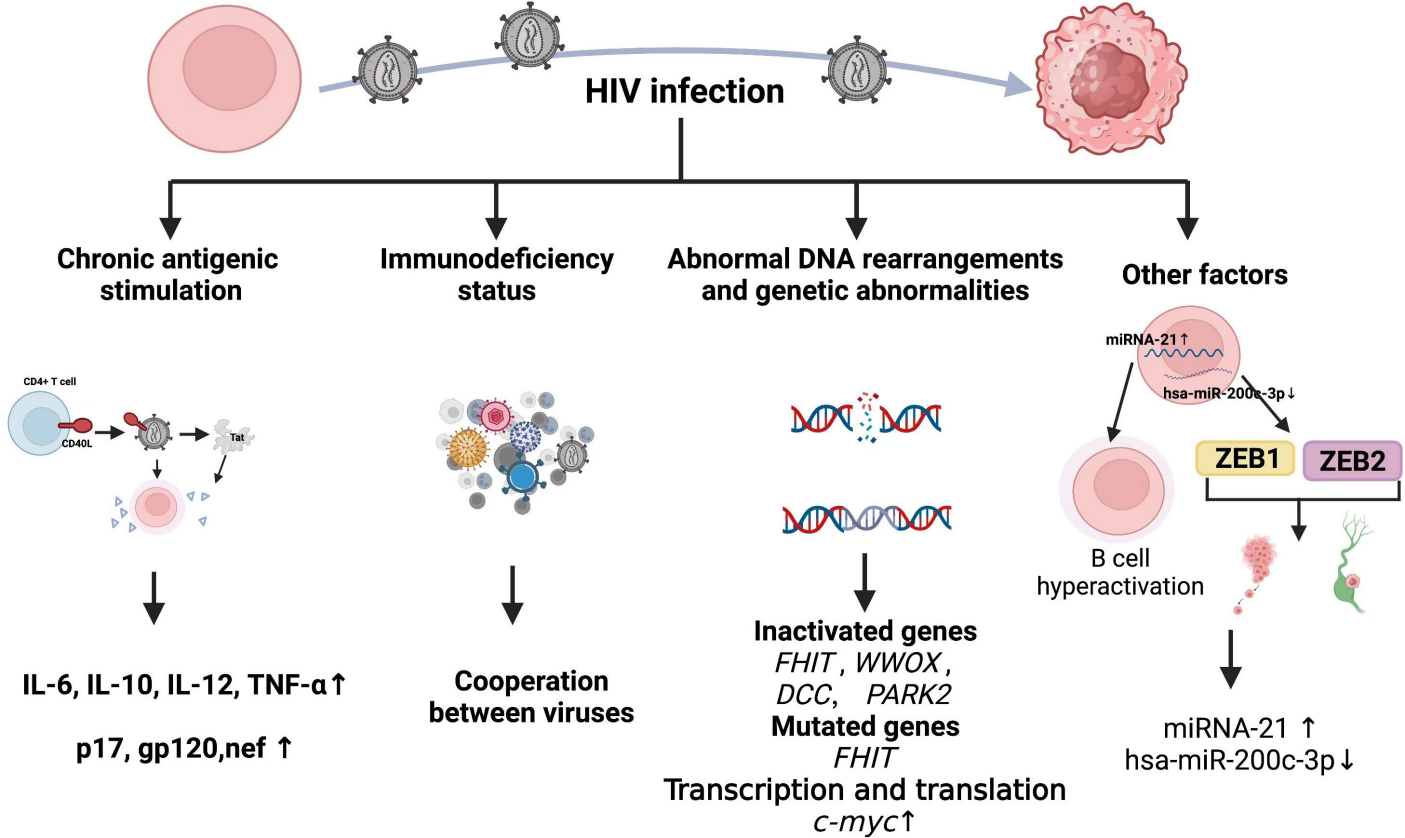
Mechanism of HIV causing lymphoma development.

That HIV causes chronic antigenic stimulation, immune dysregulation, is generally accepted. However, the high incidence of lymphoma in patients who are HIV^+^ despite the introduction of HAART suggests that incomplete immune reconstitution or factors unrelated to immune dysfunction also play causative roles. Although HIV-1 infects a subpopulation of human cells, namely CD4^+^ cells, soluble HIV-1 proteins that are detectable in serum from infected individuals invade and/or bind to receptors in uninfected cells, including B lymphocytes and endothelial cells. These proteins interfere with host gene expression and other cellular processes, ultimately leading to cellular transformation and the development of HIV-associated lymphomas. This section summarizes current mainstream views (**Figure 4**).

#### 5.2.1 Chronic antigen stimulation and cytokines

Although HIV infection is characterized by a reduction in the function or number of CD4^+^ T cells (213), the obviously increased B cell activation in HIV infection is primarily driven by the abnormal production of B cell-stimulating cytokines such as IL-6 and chronic antigenic stimulation. Elevated levels of circulating free immunoglobulin light chains in patients at increased risk of HIV-associated lymphoma might represent a marker for polyclonal B-cell activation (214). In addition, evidence indicates a skewed *IGHV* repertoire in specific HIV-NHL categories. Heterogeneous expression of *IGHV* genes in HIV-NHL might be related to specific pathways of antigenic stimulation (215).

Serum levels of IL6, IL10, C-reactive protein (CRP), soluble (s)CD23, sCD27, and sCD30 are significantly higher in patients with HIV-NHL compared with HIV^+^ or AIDS controls after adjusting for numbers of CD4^+^ T-cells (216). The CD40 ligand (CD40L) can insert itself into the surface of HIV-1 particles when budding from activated CD4^+^ T cells (217), and HIV containing CD40 ligand (CD40L) activates B cells, which leads to secretion of the cytokines, IL-6, IL-10, IL-12 and TNF-α (218), in a way that mimics physiological stimulation. The role of CD40L in cancer has been detailed in a review (219). The HIV-1 trans-activator of transcription (Tat) induces the expression of IL-6 and IL-10 at the cellular level. Findings were similar at the individual level by in transgenic mice (220), and numerous spleens from *Tat*-transgenic mice had malignant lymphomas of B-cell origin. The HIV Tat also enhances the intrinsic antibody diversification mechanism by increasing activation-induced deaminase (AID)-induced somatic mutations in the variable heavy chain (VH) region of human B cells (221), which might lead to genome-wide mutations in malignant B cells among patients with HIV.

Mice transgenic for a defective HIV-1 provirus lacking part of the gag-pol region overexpress the HIV proteins p17, gp120, and negative regulatory factor (nef), then develop B-cell lymphoma (222). This supports the pathogenic role of aberrant HIV protein and B-cell-stimulating cytokine expression during lymphoma formation. Indeed, the HIV-1 matrix protein p17 persists in the germinal center after HIV-1 drug inhibition, and its variants (vp17s) activate Akt signaling and promote the growth of transformed B cells. This protein might also upregulate LMP-1 in B lymphocytes infected with EBV, leading to lymphoma development (223). Infection HIV can directly induce lymphoma formation. The oncogenic effects of HIV-1 proteins have been reviewed in detail elsewhere (224) and are not discussed herein.

#### 5.2.2 Immunodeficiency status

For immunity, although multiple mechanisms may contribute to the development of lymphoma in HIV-infected individuals, two mechanisms appear to be involved: (1) loss of immunoregulatory control of EBV and/or KSHV; (2) chronic B-cell activation due to immune dysfunction caused by HIV infection. The cooperation of HIV, EBV, and KSHV in the pathogenesis of lymphoma and resulting microenvironmental abnormalities have been reviewed in detail elsewhere (225, 226). **Table 3** shows associations between HIV-associated lymphoma and EBV and KSHV infections. It has long been shown that B-cell activation and immature phenotypic changes *in vivo* are accompanied by polyclonal Ig production in HIV-infected individuals (228). Notably, recent studies suggest that HIV may contribute to lymphomagenesis by acting directly on B lymphocytes as a key microenvironmental factor. It is worth noting that recent studies have shown that HIV may lead to lymphomagenesis by acting directly on B lymphocytes as a key microenvironmental factor. Various HIV-encoded proteins, including gp120, may trigger and maintain abnormal activation of B cells, abnormal secretion of cytokines IL6 and IL10, and so on, which have been stated in other subsections of HIV-associated lymphomas in this paper. Perhaps it is time to revisit the second immune-related mechanism.

**Table 3:** Lymphomas in patients infected with HIV include pathological subtypes with different virus-specific associations.

HIV	EBV	KSHV
DLBCL CB	+25% (I)	–
DLBCL IB	+100% (II/III)	–
BL-plasmacytoid	+60% (I)	–
PEL and its solid variants	+90% (I)	+100
PBL of the oral cavity type	+80% (0/I)	–
Large B-cell lymphoma arising in KSHV-associated MCD	–	+100
Hodgkin lymphoma	+80%-100%(II)	–

Values indicate internal rates (%) of positivity, and infection and no infection is shown as + and –, respectively. Parentheses show latent stages of EBV. BL, Burkitt lymphoma; DLBCL-CB, diffuse large B-cell lymphoma-centroblastic; DLBCL-IB, diffuse large B-cell lymphoma-immunoblastic; MCD, multicentric Castleman disease; PBL, plasmablastic lymphoma; PEL, primary effusion lymphoma. Adopted and adapted from review by Carbon et al. (227).

#### 5.2.3 Abnormal DNA rearrangements and genetic abnormalities

Retroviruses damage DNA via various mechanisms such as genome integration, replication, inflammation, and direct interaction of viral proteins with DNA and HIV might be randomly integrated into the human genome. However, a pattern of integrated duplicated *Alu* elements and introns of Breast Cancer Gene 1 (*BRCA1*) has been identified (229) that supports the tendency of HIV-1 to integrate near the *Alu* class of human repetitive elements (230).

A genome-wide analysis of 57 HIV lymphomas found that genes associated with fragile sites such as Fragile Histidine Triad (*FHIT*; FRA3B), WW domain-containing oxidoreductase (*WWOX*; FRA16D), Deleted in Colon Cancer (*DCC*; FRA18B), and Parkinson Protein 2 (*PARK2*; FRA6E), are frequently inactivated by mesenchymal deletions in HIV-NHL, and that the prevalence of *FHIT* alterations is significantly higher in HIV-DLBCL (231). Among these, *FHIT*, *WWOX* and *DCC* are tumor suppressor genes that are frequently inactivated in various human malignancies (232–234). Thus, HIV might act directly at the genomic level to promote the pathogenesis of HIV-NHL, and this translational effect is partially independent of the expression of viral oncogenes. Human immunodeficiency virus induces *c-myc* dysregulation in B cells, and levels of viral RNA and *myc* expression correlate (235). Expression of the highly oncogenic transcription factor *c-myc* is enhanced at the transcriptional and translational levels in the presence of HIV-1 Tat protein (236).

#### 5.2.4 Other factors

Viruses and their components manipulate the expression of host miRNAs and play important roles in cancer pathogenesis. Hsa-miR-200c-3p is significantly downregulated in HIV-associated BL, and the zinc finger E-box binding homeobox epithelial-mesenchymal transition (EMT) transcription factors ZEB1 and ZEB2 are upregulated and actively help to promote tumor metastasis and invasion (237). Moreover, miRNA-21 is significantly elevated in peripheral B cells of patients infected with HIV, suggesting that it might contribute to the maintenance of B cell hyperactivation (238). A proteomic analysis of plasma proteins from AIDS-NHL recently identified 20 host proteins and a set of protein combinations that might serve as biomarkers for the pathogenesis of AIDS-NHL (239). This indicates a new direction towards a better understanding of the pathogenesis of HIV lymphoma.

## 6 Kaposi sarcoma-associated herpes virus

This virus (human herpesvirus-8, HHV-8) is the causative agent of Kaposi sarcoma (KS) and is associated with the lymphoproliferative primary exudative lymphoma (PEL) and the plasmablastic form of MCD (240, 241). The other types of lymphoma associated with KSHV are KSHV-positive large B-cell lymphoma not otherwise specified (NOS) and GLPD. The geographic distribution of KSHV is variable, with the prevalence of infections being highest in sub-Saharan Africa (seropositivity > 50%), intermediate in Mediterranean, Middle Eastern, and Caribbean countries (seropositivity 20%-30%), and lowest in Asia, Europe, and the USA (seropositivity < 9%) (242). At present, the transmission route of KSHV is not completely clear, but it is believed that the infection mainly occurs through salivary transmission (243). Several studies have shown that KSHV can infect almost any type of cells, including epithelial cells, monocytes, macrophages, dendritic cells, T cells and fibroblasts. (243) Lymphoproliferative primary exudative lymphoma is a rare HIV-associated non-Hodgkin lymphoma (NHL) that accounts for ~ 4% of all HIV-associated NHL. This type of lymphoma tends to locate in the pleural space, pericardium, and peritoneum. It is morphologically variable with an empty lymphocyte immunophenotype and evidence of KSHV infection (244). It is aggressive, rapidly progressive, and is associated with high mortality rate; the average survival of patients with PEL is 2- 6 months (245).

### 6.1 Kaposi sarcoma-associated herpes virus structure

The KSHV genome consists of linear double-stranded DNA that is cyclized during latent infection. It contains a unique coding sequence of ~ 140 kb flanked by 25-30 kb repetitive terminal repeats (246). The life cycle of KSHV is biphasic, with consecutive latent and lytic replication phases (247, 248), each of which has a unique gene expression profile like EBV (249). The viral oncoproteins, KSHV latency-associated nuclear antigen (LANA; ORF73), vCyclin, and *l*atent viral FADD-like interleukin-1-converting enzyme (FLICE) inhibitory protein (vFLIP) are encoded by KSHV during the latent phase, whereas KSHV G protein-coupled receptor (vGPCR), viral B cell lymphoma 2 (vBcl-2), vIL-6, viral IFN regulatory factor 1(vIRF) 1/vIRF 3, K1, K15, and viral protein kinase (vPK) (250) are encoded during the lytic phase.

### 6.2 Carcinogenic mechanisms

KSHV has evolved to produce a large number of viral gene products that intricately subvert normal cellular pathways. The proteins encoded by KSHV that are thought to have transformative and oncogenic properties include latent proteins, which increase the survival and proliferation of infected cells, and lytic proteins, which are thought to mediate tumor growth. Due to space constraints, this section only summarizes the main mechanisms.

#### 6.2.1 viral proteins

##### 6.2.1.1 LANA

The mechanisms underlying KSHV carcinogenesis remain unclear. Analysis of infected cells by immunofluorescence and immunohistochemistry confirmed that LANA is one of the latent proteins consistently present in all KSHV-infected tumor cells of Kaposi’s sarcoma, PEL and MCD. (251) As a multifunctional protein, LANA is involved in the regulation of transcription, chromatin remodeling, exome maintenance, DNA replication, and the control of latency and lytic phase reactivation. In addition, LANA is also involved in cell cycle regulation, which has been described in the review by Wei et al. (251) LANA binds to and inactivates the tumor suppressor proteins TP53 and retinoblastoma (RB1), thereby regulating cell growth. (252) LANA expression also affects MYC levels by binding to the negative regulator GSK-3β and thus promotes lymphomagenesis. (253) Based on current knowledge, LANA appears to provide the basis for at least the formation of KSHV-associated lymphomas.

##### 6.2.1.2 Viral cyclin

Viral cyclin (ORF72) is a viral homolog of cell cycle protein D (254) which plays an important role in lymphangiogenesis via several functions. Physiologically, cyclin D forms a complex with cyclin-dependent kinase (CDK) and CDK4 that phosphorylates retinoblastoma protein (Rb) and leads to the release of E2F transcription factors (255). The KSHV vCyclin interacts with CDK6 to promote cell cycle progression (256, 257). Moreover, the vCyclin/CDK6 complex can phosphorylate nuclear phospholipid histone chaperones, leading to genomic instability (258).

##### 6.2.1.3 vFLIP

vFLIP is the viral homologue of cellular FLIP. Transgenic mice expressing vFLIP exhibit B cell transdifferentiation and acquire the ability to express histiocyte/dendritic cell markers (259). These mice have hematological properties typical of PEL and MCD. Previously, it has been found that vFLIP prevents apoptosis by up-regulating NF-κB. (260) In addition, the study of Lee et al. demonstrated that vFLIP can protect cells by preventing autophagy to further maintain latency. (261).

##### 6.2.1.4 vIL-6

vIL-6 is the viral homologue of hulL-6, and immunohistochemistry has shown that it is expressed in variable proportions in KSHV^+^ lymphoproliferative lesions. (251) One characteristic of KSHV-driven PEL is elevated serum human IL-6 (hIL-6) levels. Notably, v-IL6 can replace hIL-6, activating it constitutively via the rat sarcoma/mitogen activated protein kinase (Ras/MAPK) and JAK/STAT pathways (262).

#### 6.2.2 miRNAs

KSHV miRNAs are generated from 12 pre-miRNA transcripts in the latency region, ultimately producing at least 17 mature miRNAs. (263) The biogenesis of KSHV miRNAs and their role in the development of KSHV-associated malignant tumors has recently been described in detail. (242, 264) Among the large number of miRNAs encoded by KSHV, KSHV-miRNA-K11 compares particularly because it shows significant homology to cellular miRNA-155. (265) MiRNA-155/bic overexpression can be observed in many human B-cell lymphomas, (266) and B-cell lymphomas can be induced in mice. (267).

## 7 Conclusions

The main aspect of virus-driven lymphangiogenesis initially focuses on the direct transforming activity of a single viral oncogenic product. However, cooperation among different viruses also plays crucial roles in the development, survival, and dissemination of lymphoid malignancies. Therefore, many studies have targeted relationships among the microenvironment, oncogenesis, tumor growth, and dissemination. How EBV and KSHV support each other in terms of persistence and lymphangiogenesis has been explained in recent reviews (268), (269). A relationship between EBV and HCV replication markers has not been identified in patients with AIDS (270), which is in contrast to other known coinfections. Indeed, HCV and HBV co-infection inhibits HCV replication, whereas HCV and HIV co-infection stimulates HCV replication and exacerbates HIV-associated immunosuppression, and EBV and HIV co-infection stimulates HIV replication in CD4T cells (271, 272). All of these complicate understanding the mechanisms through which co-infection causes carcinogenesis. To further elucidate and characterize the mechanisms of viral induction of lymphoma is a considerable challenge that will require an integrated multidisciplinary approach involving epidemiologists, molecular biologists, and immunopathologists.

## Author contributions

YZ: Writing – original draft, Writing – review and editing, Validation. WG: Investigation, Writing – review and editing. ZZ: Investigation, Writing – review and editing. OB: Supervision, Writing – review and editing.
